# Vocal Features of Song and Speech: Insights from Schoenberg's Pierrot Lunaire

**DOI:** 10.3389/fpsyg.2017.01108

**Published:** 2017-07-11

**Authors:** Julia Merrill, Pauline Larrouy-Maestri

**Affiliations:** ^1^Music Department, Max Planck Institute for Empirical Aesthetics Frankfurt, Germany; ^2^Institute of Music, University of Kassel Kassel, Germany; ^3^Neuroscience Department, Max Planck Institute for Empirical Aesthetics Frankfurt, Germany

**Keywords:** vocal expression, sprechgesang, categorization, speechsong, voice

## Abstract

Similarities and differences between speech and song are often examined. However, the perceptual definition of these two types of vocalization is challenging. Indeed, the prototypical characteristics of speech or song support top-down processes, which influence listeners' perception of acoustic information. In order to examine vocal features associated with speaking and singing, we propose an innovative approach designed to facilitate bottom-up mechanisms in perceiving vocalizations by using material situated between speech and song: Speechsong. 25 participants were asked to evaluate 20 performances of a speechsong composition by Arnold Schoenberg, “Pierrot lunaire” op. 21 from 1912, evaluating 20 features of vocal-articulatory expression. Raters provided reliable judgments concerning the vocal features used by the performers and did not show strong appeal or specific expectations in reference to Schoenberg's piece. By examining the relationship between the vocal features and the impression of song or speech, the results confirm the importance of pitch (height, contour, range), but also point to the relevance of register, timbre, tension and faucal distance. Besides highlighting vocal features associated with speech and song, this study supports the relevance of the present approach of focusing on a theoretical middle category in order to better understand vocal expression in song and speech.

## Introduction

Language and music processing have been the subject of comparison for decades. Patient studies have revealed a dissociation between pitch processing in language and music (e.g., Peretz and Coltheart, 2003), but studies on music expertise and its transfer in the language domain support shared processes (e.g., Patel, 2008). While conclusions are still being discussed, researchers agree that the comparison of sung material to speech is interesting due to their shared properties. Indeed, both signals are produced with the same instrument (Titze, 1994, 2008; Sundberg, 2013) and share comparable structure (i.e., modulation of a monophonic acoustical signal over time, presence of lyrics/articulatory features, and following of syntactic rules; Koelsch, 2011). However, the comparison of speech and song requires a deep understanding of their respective characteristics and therefore a clarification of what makes a vocalization stand out as speech or song.

### Production of vocal features in speech vs. song

Vocal production basically consists of oscillating movements of the vocal cords, initiated and sustained by the air stream from the lungs (Titze, 1994, 2008). Pitch control in speech and song is achieved by the combination of tension and geometry of muscles in the larynx and sub-glottal pressure (Sundberg, 2013). Also, the vocal cavity and its geometry serve the function of a filter, which produces frequency bands of enhanced power in the vocal sound (Fant, 1960). However, sound modulations have been described as being different depending on the purpose of the vocalization (i.e., singing vs. speaking).

Regarding the pitch dimension, the German linguist Sievers (1912) described the difference between music and speech as early as 1912: “Music works mainly with fixed tones of steady pitch, speech moves mainly in gliding tones that rise and fall from one pitch to the other within one and the same syllable. Speech in particular is not bound to discrete pitches and intervals of musical melodies: it knows approximate tone levels only.” A 100 years later, this is the main difference described in current publications (e.g., Patel, 2008; Zatorre and Baum, 2012). When investigating the acoustical features in song and speech production, the patterns that most stand out in visual inspection of the spectrogram are the straight, horizontal lines in song when holding a tone and the ups and downs of fundamental frequency (F0) when examining speech signals (for a good visualization, see Zatorre and Baum, 2012). That means, when investigating the acoustical features of the pitch patterns (pitch contour, melodies) in music and speech, a fundamental difference remains in the gliding, continuously changing pitch in speech and the discrete pitch in music; and while musical melodies are built around a stable set of pitch intervals (according to Western tonal music), spoken prosody is not (e.g., Patel, 2008). The variations of pitch in music are rather fine grained (one or two semitones; Vos and Troost, 1989), while in speech they can be rather coarse, for example, up to more than 12 semitones (Patel et al., 2008). Note that music seems to reflect patterns of durational contrast between successive vowels in spoken sentences, as well as patterns of pitch interval variability in speech (Patel et al., 2006) but, in singing, vowels are typically elongated to achieve the rhythm dictated by the musical text and to convey the pitch assigned to a specific syllable.

Besides the numerous studies comparing pitch production in speech and song, few studies have investigated the differences between speech and song with regard to other features such as vocal quality (Lundy et al., 2000; Livingstone et al., 2014; Gafni and Tsur, 2015), tension or articulation. In order to describe the peculiarities in spoken and sung signals (by applying acoustical analyses to the vocalization), a crucial step consists of understanding what is relevant to listeners when perceiving vocalizations as either speech or song.

### Perception and evaluation of vocal features in speech vs. song

On a perceptual level, clear speech and song stimuli can easily be distinguished by listeners, while the classification of ambiguous stimuli into the categories of song and speech is an individually varying process (Merrill et al., 2016). The distinction and classification of the two modes of phonation may result from the development of expectations about each domain. Indeed, the functions/contexts of these two activities are clearly distinguished from an early age (McMullen and Saffran, 2004). More generally, categorization is facilitated (if not driven) by top-down cognitive processes that constrain the listener's perception and therefore judgment. The phenomenon of categorical perception has been extensively studied on several perceptual dimensions (Harnad, 1987; for a review on the phenomenon, see, Goldstone and Hendrickson, 2010), such as categorization of phonemes (Liberman et al., 1957), categorization of environmental sounds (Giordano et al., 2014), prosodic contour (Sammler et al., 2015), or speaker gender (Latinus et al., 2013). The existence of distinct categories relative to speech and song is also supported by the well-known speech-to-song illusion (Deutsch, 2003; Deutsch et al., 2008, 2011; Tierney et al., 2013; Jaisin et al., 2016). By listening to a spoken phrase several times, listeners perceive the phrase as sung. This simple and compelling experiment suggests that the categorization of a performance as speech or song does not rely on the acoustical characteristics (always the same) but rather on the repetition effect leading to the reinterpretation of the material as musical (Falk et al., 2014). However, when listening to a single sound or isolated phrase (i.e., not repeated and without previous exposition to the phrase), listeners are able to detect a clear spoken or sung utterance (Merrill et al., 2016) and therefore rely on acoustical parameters of the signal itself or on perceptual impressions.

To the best of our knowledge, the vocal features underlying the perception of a vocalization as song or speech remain unclear. One reason might be due to the proximity/similarity of several acoustic features usually examined (Tierney et al., 2013), which could overshadow the subtle differences in other acoustical features perceived by listeners. Another reason might be the lack of adequate tools to examine listeners' perception of vocal features in both sung and spoken material. Some questionnaires are specific to singing (e.g., Henrich et al., 2008), while others are specific to speech (e.g., the Geneva Voice Perception Scale, Banziger et al., 2014), making difficult a direct comparison of the impressions of spoken and sung vocalizations. A questionnaire was designed to capture the vocal-articulatory impression of vocal expression on many levels (Bose, 2001, 2010), and can be used in different contexts, such as describing adults as well as children's speech (Bose, 2001), artistic and emotional speech (Wendt, 2007). Items are grouped into different major categories with regard to pitch, loudness, sound of voice and their subsequent modifications, articulation and complex descriptions of vocal expression such as mode of phonation (speaking, speechsong, singing etc.), rhythm, or tempo. By relying exclusively on listeners' perception, this tool overcomes the limits of acoustical analyses (i.e., acoustical differences between sound signals are not necessarily relevant to listeners) and was used for the first time to examine the vocal features leading to the perception of speech and song.

Another important reason concerns the choice of material to examine. In order to focus on the perceptual impressions of listeners, particular attention must be paid to limit the top-down cognitive processes behind categorization of vocalization (Falk et al., 2014; Margulis et al., 2015; Vanden Bosch der Nederlanden et al., 2015; Jaisin et al., 2016). Schematically, human perception is influenced by the existence of preset categories and expectations based on these categories (i.e., top-down processes) as well as the physical features of the stimuli (i.e., bottom-up processes). As the same principle applies to vocalization, examining the perception of acoustic features or listeners' impressions requires the use of material which is neither typical to the language nor to the music domain. Unfortunately, speech and song are rarely melded in a single performance, except in art forms such as poetry or rap music (Wallin et al., 2000; Fritz et al., 2013) or in a musical phenomenon called “speech-song” (Stadlen, 1981). Speechsong (or “Sprechgesang” in German) can be described as an expressionist vocal technique resembling an intermediate state between speech and song. By its nature, speechsong seems to be an adequate candidate to suppress the categories “speech” and “song,” and therefore allows for examination of listeners' perceptual impressions.

### The case of speechsong

Building partly on earlier forms combining prosodic aspects of speech and music, such as the recitative and the melodrama, the form of speechsong in Western Art Music has been around for over 100 years, and its reception has fallen very far from any other type of vocal performance. The piece used in the current study is the representative of speechsong at the brink of modernity: Three times Seven Poems from Albert Giraud's “Pierrot lunaire” op. 21 for speaking voice and chamber orchestra by Arnold Schoenberg, written in 1912. The music lacks a tonal center, called atonal, and is a highly discussed piece by musicologists and performers alike. The 21 poems are composed into 21 short pieces. Instructions on how to perform Pierrot can be found in its preface and are represented in the musical score by a special musical symbol for the vocal part. The most important instruction was that the melody “should definitely not be sung” but had to be “transformed into a speech melody” (Schoenberg, 1914). Furthermore, “the difference between a sung pitch and a spoken pitch should be clear: a sung pitch is held and does not change, whereas a spoken pitch is intoned, then left by rising or falling in pitch” (Schoenberg, 1914). It is also interesting that Schoenberg did not only differentiate his notated vocal part from singing, but also from normal speaking: “The difference between normal speaking and speaking as part of a musical structure should be clear” (Schoenberg, 1914). It is also of note that he instructed that the notated rhythm be precise. Hence, the performer has the task of achieving the impression of speaking by following a notated rhythm with long notes, fixed pitch and a pitch range covering 2.5 octaves (from E flat 3 to G sharp 5). All this together constituted significant challenges for the performer, which were further addressed by the composer later: “The pitches in Pierrot depend on the range of the voice. They are to be considered ‘good’ but not to be ‘strictly adhered to’. […] Of course, the speaking level is not enough. The lady must just learn to speak in her ‘head voice’…” (Schoenberg, 1923). His idea of the vocal part was not limited to melodic accuracy though, as the overall vocal expression was considered important, such as “to capture the light, ironical, satirical tone […] in which this composition was originally conceived” (Schoenberg, 1940). Taken together, a degree of freedom is given with regard to the pitches and performers regularly narrow down the compass of their voice and produce relative intervals between pitches (Cerha, 2001, p. 67). This has been evidenced by empirical investigations about Pierrot performances, focusing on the melodic accuracy with regard to the musical score, i.e., counting exact and deviating pitches and correct and incorrect pitch interval relations (Heinitz, 1925), and making assumptions about the relationship between the notated melodies and linguistic and emotional prosody (Hettergott, 1993; Rapoport, 2004, 2006).

Our main objective consists of clarifying the features of vocal expression associated with speaking and singing on an impressionistic level. We propose an innovative approach that aims at minimizing the top-down processes influencing listeners' perception of auditory information. Concretely, listeners are asked to evaluate various interpretations of a representative speechsong composition with an extensive questionnaire. It is expected that such material will not clearly be considered speech or song. To examine the relationship between evaluations of the mode of phonation (i.e., speaking and singing) and a variety of features, including not only features of the pitch domain but also of vocal sound and articulation, voice experts were chosen as participants. By achieving evaluations at this detailed level, we aim at clarifying what causes a vocalization to be perceived as speech or song.

## Materials and methods

### Ethics statement

All experimental procedures were ethically approved by the Ethics Council of the Max Planck Society, and were undertaken with written informed consent of each participant.

### Participants

The raters were 25 university students from the field of speech science and phonetics (20 female, mean age = 23.3 years, *SD* = 1.14). They were equally trained in the auditory description of voices and in using different vocal assessment scales, and most importantly, in the questionnaire used in the current study. Their expertise was verified by taking their study program into account, which demonstrates their level of training, proven by corresponding periodical and final examinations (e.g., Bachelor's degree). The raters were all German native speakers. All of them had had singing training and/or played an instrument (range 4–18 years of training, *M* = 6.76) and 14 of them were still practicing music at the time of the experiment. One participant had a master's degree in Music (main instrument was the trumpet), one in acting, one in classical singing, five participants were trained speech therapists. Note that participants were unfamiliar with the piece under study prior to the testing session, which ensures comparable knowledge among participants.

### The musical piece

From the composition Pierrot lunaire, the piece No. 7 “The Sick Moon” was chosen because of the sparse accompaniment, which is the flute only, so that the voice could be heard clearly. The lyrics were in the original German. The chosen excerpt ended with the first stanza. The excerpts were between 35 and 55 s long, depending on the chosen tempo by the performer. The pitch range of the excerpt is from D 4 to E flat 5 and the dynamic range is small, ranging from pianissimo to piano with several short crescendi and a decrescendo.

### The recordings

Table 1 shows the 20 interpretations used in the study. The recordings range from 1940 to ca. 2010, representing a wide range and great variety of interpretations. The earliest one was conducted by Schoenberg himself, followed by recordings from his friends and colleagues Josef Rufer and René Leibowitz (1949 and ca. 1954, respectively). Pierre Boulez recorded the piece three times with a different performer each time.

**Table 1:** List of recordings.

**Conductor**	**Performer**	**Year of recording**	**Label, code**
Angius, Marco	Rado, Livia	ca. 2010	STR33962
Atherton, David	Thomas, Mary	1973	Decca 4256262
none	Goltz, Jennifer	ca. 2007	MSR Classics MS1208
Boulez, Pierre	Minton, Yvonne	1977	B00000281B
Boulez, Pierre	Pilarczyk, Helga	1961	WER6778-2
Boulez, Pierre	Schäfer, Christine	1997	E4576302
Ceccanti, Mauro	Bergamasco, Sonia	1997	Arts 47389-2
Craft, Robert	Silja, Anja	ca. 2000	Naxos 8557523
de Leeuw, Reinbert	Sukowa, Barbara	1988	Koch Schwann 310117
Engelen, Robin	Janssen, Jaqueline	2003	FUG504
Gould, Glenn	Rideout, Patricia	1974	74645266428
Gourzi, Konstanzia	Doufexis, Stella	ca. 2007	NEOS10709
Herreweghe, Philippe	Pousseur, Marianne	1991	HMA1951390
Leibowitz, René	Semser, Ethel	ca. 1954	BAM LD 016
Rattle, Simon	Manning, Jane	1977	CHAN6534
Rufer, Josef	Burmester, Irmen	1949	Audite 21.412
Schönberg, Arnold	Stiedry-Wagner, Erika	1940	CBS MPK 45695
Sinopoli, Giuseppe	Castellani, Luisa	1997	Teldec 3984-22901-2
Weisberg, Arthur	DeGaetani, Jan	1970	H-71251
Zender, Hans	Kammer, Salome	1994	MDG 613 0579-2

*List of recordings with conductor, performer, year of recording, label and code*.

### The questionnaire on vocal-articulatory expression

The questionnaire was developed by speech scientists (in German, Bose, 2001, 2010) as a tool for describing the vocal-articulatory expression in (German) speech. As illustrated in **Table 3**, the tool is set up to combine features that represent auditory impressions, not acoustic measures. Items are grouped into different major categories (*N* represents the number of items selected for the current study) with regard to pitch and its modifications (*N* = 4), loudness (*N* = 1), sound of voice and its modifications (*N* = 14), articulation (*N* = 3) and complex descriptions of vocal expression (*N* = 4), such as mode of phonation (sung or spoken) or rhythm. The modifications are described by means of the variability (e.g., for pitch inflected or monotone), the range (wide or narrow), and the changes between tones or syllables (sudden or continuous). Items pertaining to the sound of voice are manifold, e.g., faucal distance describes a wide or constricted pharynx, the sound of the onsets and offsets of the voice are described with hard and soft (for detailed description of the features and more extensive background information on the questionnaire, please refer to Bose, 2001). For the current study, the profile was slightly adapted to the musical piece under study. An item about the register and blending of the voice and the flute was added, while the category of loudness was reduced down to its range since the possibilities were limited in the recordings used. Overall, the questionnaire consisted of 27 items: 20 items were specific to vocal expression (5-point scale; six items on a 3-point scale, see Table 2), one item concerned the flute and the voice (5-point scale). Note that the use of such a questionnaire requires experience in voice diagnostics and/or familiarity with the specific vocabulary and dimensions to evaluate. Because lay people cannot differentiate, for example, between a rough and a breathy voice (Kreiman and Gerratt, 1998), the present study focuses on the evaluation by voice experts.

**Table 2:** Questionnaire on vocal-articulatory expression.

**Categories**	**Features**	**Characteristics**	
Pitch	Average pitch	Low	High
	Pitch variability	Inflected	Monotone
	Pitch range	Wide	Narrow
	Pitch changes	Sudden	Continuous
Loudness	Loudness range	Wide	Narrow
Sound of voice	Resonance	Full	Thin
	Timbre	Dark	Bright
	Faucal distance	Wide	Constricted
	Vocal onset, offset	Soft	Hard
	Modifications: ~Variance	Varying	Constant
	~ Range	Wide	Narrow
	~ Changes	Sudden	Gradual
	Register	Chest voice	Head voice
	Noisiness	Breathy	
		Pressed	
		Creaky	
		Harsh	
	Other modulations	Vibrato	
		Tremolo (tremble)	
Articulation	Precision of articulation	Precise	Imprecise
	Vowel duration	Lengthened	Shortened
	Consonant duration	Lengthened	Shortened
Other	(Phonation) tension	Tense	Relaxed
	Mode of phonation	Speaking	Singing
	Rhythm	Staccato	Legato
	Overall tempo	Slow	Fast
	Blending of voice + flute	Dis-harmonic	Harmonic

*The five rational categories (1st column) pitch, loudness, sound of voice, articulation and other are divided into several features (2nd and 3rd column) describing the vocal expression, in columns #3 and #4 are the bipolar characteristics. Features on noisiness and other modulations are unipolar*.

In the present study the questionnaire was used to examine voices in an artistic/musical context; the midpoint of the rating scale, which usually describes a neutral midpoint, was now representing the perception of a feature being “just about right” (JAR) to account for the listeners' expectations of the vocal part. If a feature was perceived as “not right,” then the rater could choose the direction in which it deviated from JAR. In the case of the perceived “average pitch” of a voice, it could be “too high” and “much too high” or “too low” and “much too low.” The rating scale was adapted from studies in consumer product testing (Popper et al., 2004; Popper and Kroll, 2005) and has been used to evaluate piano performances (Kroger and Margulis, 2017). As can be seen in Table 2, most items were bipolar (e.g., the average pitch can be high or low), the items on noisiness and modulations of the voice were unipolar, e.g., the vibrato could be rated as “just about right” or not. These items were only rated upon when present in the voice.

The JAR-scale can be coded in two different ways: A coding from 1 to 5 takes the bipolar scale into account; A coding accumulating the deviations from JAR allows focusing on the “JAR-ness,” i.e., a feature being either JAR (value 0) or non-JAR with “too X” (1) and “much too X” (2).

Besides these items of vocal expression, two general questions were proposed at the beginning of the questionnaire: overall liking and adequateness. Liking ratings are based, for instance, on the individual musical preferences and spoken arts perception, or on liking of the voice etc. Adequateness ratings, on the other hand, were dependent on the background information provided to the participants about Pierrot (see below). The questionnaire was concluded with two additional questions: “Do you consider the interpretation as coherent (additional meaning of the German phrase might be expressed by ‘harmonious’ and ‘cohesive’)?” (yes/no) and “What is the profession of the performer?” (Singer, speaker or actress). The ratings for profession were challenging (i.e., both “speaker” and “actress” boxes were often ticked together in the present study) and might be more informative when coded as binary variables, i.e., singer and actress. Coherence is about the overall impression of the performance (including voice and flute) and reflects the listener's expectations regarding the interpretation of the piece.

### Procedure

Prior to the listening task, an introduction to the composer and the piece under study was presented by the experimenter, including Schoenberg's dates of birth and death and selected historical-musicological background information, i.e., his role in shifting from tonality to atonality in music as well as the importance of Pierrot with regard to the extraordinary use of the vocal part. With regard to the musical piece, the preface to Pierrot was presented, as well as selected quotes from Schoenberg (all presented in the Introduction “The case of speechsong”). The goal was to form a knowledge basis for the raters who were partly not familiar with Schoenberg and not at all familiar with the piece Pierrot lunaire. Also, the musical score of the beginning of the first piece (i.e., not the chosen piece) was presented in order to illustrate the peculiar musical notation of the vocal part. The participants also listened to four very different interpretations beforehand (which were not included in the respective experimental session) to get an idea on the range of possible interpretations, and to get to know the chosen piece, “The Sick Moon.”

During the rating sessions, excerpts were played three times, in a pseudo random order, counter balanced between two groups. After the first presentation, participants rated the overall liking and the adequateness. Then, while listening to the piece two more times, they rated the items on vocal expression as well as the additional questions. Participants were informed that they do not have to fill out the items in the order presented. Participants rated all 20 recordings in two sessions (70–90 min each).

### Statistical analyses

Since both the musical piece and the extraordinary vocal performance were unfamiliar to listeners, inter-rater agreement at the level of features of vocal expression was estimated following the procedure described in Larrouy-Maestri et al. (2013). Pairwise Spearman coefficient correlations between the 25 participants (rating 20 items for each of the 20 interpretations) were computed. On the basis of the correlation matrix, the proportion of significant correlations as well as their median correlation coefficient are reported. The agreement of the more general questions was investigated following similar procedures, using Spearman coefficients for the two scales (i.e., liking and adequateness) and using the phi coefficient for the two categorical variables (i.e., cohesion and profession). Differences in agreement level depending on the general question were tested with Chi-square tests.

Next, the relationship between the features and general questions was examined by means of linear regression analyses for liking and adequateness ratings and logistic regression analyses for coherence and profession ratings (using the JAR-ness scale 0–2). Each analysis was carried out with the 20 items as potential predictors. Spearman coefficient correlations were computed between the “influential” items (using the 1–5 scale) and the general questions. For instance, if the coefficient correlation did not reach significance, low liking is related to high scores in both directions (e.g., average pitch of the voice being “too low” or “too high”). If the coefficient correlation was significant and positive, low liking was associated with the item on the left side of the scale (see Table 2). In the case of a negative correlation coefficient, low liking was associated with the item on the right side of the scale.

Finally, the relation between the mode of phonation and the other items in the questionnaire was examined with Spearman correlations using Bonferroni adjusted alpha levels of 0.003 in order to investigate features related to performances being perceived as “too (much) sung.” Correlations were performed on the values 1, 2, 4, and 5, to focus on the direction of the relations. Also, a particularly relevant feature was investigated further with a Chi-square test. Here, the ratings were accumulated such that the mean was taken from each direction of the rating scale to only have one value representing song and one representing speech. Statistical analyses were performed using IBM SPSS Statistics 22.

Some items were only ticked occasionally and were not further analyzed, i.e., the items on noisiness and modulations (overall *N* ≤ 14), as well as the item about the blending of flute and voice as almost all ratings were toward “disharmonic.”

## Results

### Inter-rater agreement

With regard to the features of vocal expression, the median correlation coefficient for the full scales (1–5) was *r* = 0.339 (*SD* = 0.072). Despite the moderate median correlation, all the combinations reached the significance level of *p* < 0.05. The proportion of positive and significant correlations (for the liking and adequateness questions) and phi coefficients (for the coherence and profession questions) are reported in Table 3.

**Table 3:** Inter-rater agreement.

	**Coherence**	**Adequateness**	**Liking**	**Profession**
Proportion of positive and significant correlations	8.33%	20.00%	32.67%	39.00%
Median correlation coefficient	0.519	0.530	0.565	0.655
Mean correlation coefficient	0.548	0.561	0.575	0.646
*SD*	0.107	0.098	0.092	0.126

*Estimation of the agreement between the 25 raters (proportion of positive and significant Spearman correlation coefficient/phi coefficients, median correlation coefficient, mean correlation coefficient and standard deviation) when rating the general liking of each piece, the adequateness, the coherence of the interpretations, and the profession of the performers*.

In contrast to the high agreement of the participants when rating features of vocal expression (i.e., 100% of the correlations were positive and significant), the proportion of agreement regarding general liking, adequateness, coherence, and profession of the performers, was much lower (see Table 3). Chi-square tests confirmed the difference in agreement level depending on the general question proposed. Agreement of participants increased significantly following the order of Table 3 (left to right): Coherence – Adequateness [$\chi_{\left(4\right)}^{2}$ = 313.55, *p* < 0.001], Adequateness – Liking [$\chi_{\left(4\right)}^{2}$ = 326.10, *p* < 0.001], Liking – Profession [$\chi_{\left(4\right)}^{2}$ = 312.176, *p* < 0.001].

Importantly, despite the relatively low agreement on the general level, it was observed that when participants provided similar ratings, the median or mean correlation coefficients were “high” (>0.5) and presented low variability. In other words, few participants shared the same opinion, but when it was the case, they strongly agreed regarding the liking, adequateness, coherence and the profession of the performer.

### Relationship between the features and general questions

#### Liking

Regarding participants' liking, a significant regression equation was found, *F*_(20, 433)_ = 19.647, *p* < 0.001. As can be seen in Table 4, several items contributed in explaining 47.6% of the variance of the Liking ratings. According to the Spearman correlations, low liking was associated with a high average pitch, a thin and hard sound of voice, a constricted pharynx and lengthened vowels. Low liking was also associated with pitch variability and changes, the variance of the sound and mode of phonation. However, the ratings of these items were evenly dispersed on both sides of the scale, e.g., “too sung” and “too spoken” were both associated with low liking. More generally, it is of note that the performances were not particularly liked, with a median of 4 on the scale (IQR = 1–6).

**Table 4:** Regression analyses.

		**Liking**			**Adequateness**			**Coherence**			**Profession**		
				**Direction**			**Direction**			**Direction**			**Direction**
		**Beta**	***p*-value**	***r***	**Beta**	***p*-value**	***r***	**Beta**	***p*-value**	***r***	**Beta**	***p*-value**	***r***
**(Constant)**			**0.000**			**0.000**			**0.000**			**0.035**	
Pitch	Average pitch	−0.219	0.000	−0.173^*^	−0.170	0.001	−0.076	−0.002	0.994		0.105	0.654	
	Variability	−0.183	0.000	0.012	−0.093	0.075		0.596	0.016	−0.034	−0.065	0.785	
	Range	0.008	0.844		−0.062	0.229		0.589	0.020	0.048	0.409	0.110	
	Changes	−0.090	0.039	0.063	−0.139	0.007	−0.167^*^	0.020	0.932		0.082	0.713	
Loudness	Range	0.070	0.087		0.039	0.428		0.100	0.657		0.219	0.326	
Sound of	Resonance	−0.133	0.003	−0.432^*^	−0.073	0.164		0.099	0.696		0.359	0.141	
voice	Timbre	−0.034	0.415		−0.010	0.837		0.686	0.007	0.183^*^	−0.085	0.704	
	Faucal distance	−0.098	0.023	−0.340^*^	0.042	0.413		0.774	0.001	0.308^*^	0.226	0.333	
	Sound	−0.103	0.016	−0.162^*^	0.085	0.091		−0.009	0.972		0.569	0.020	0.101^*^
	Variance	−0.137	0.002	−0.002	−0.010	0.843		0.166	0.487		0.100	0.671	
	Range	0.0024	0.560		0.026	0.602		−0.431	0.103		0.245	0.319	
	Changes	−0.042	0.351		−0.025	0.641		0.391	0.121		0.342	0.168	
	Register	−0.039	0.345		−0.070	0.158		0.400	0.083		−0.182	0.398	
Articul.	Precision	−0.008	0.842		−0.174	0.000	−0.161^*^	0.092	0.689		−0.329	0.149	
	Vowel duration	−0.106	0.006	0.272^*^	0.033	0.467		0.629	0.006	−0.205^*^	−0.047	0.823	
	Cons. duration	−0.004	0.926		−0.093	0.051	0.159^*^	0.126	0.628		0.093	0.719	
Tension		0.010	0.798		0.000	0.992		0.410	0.086		0.241	0.309	
Mode of phonation		−0.113	0.002	0.076	−0.191	0.000	−0.080	0.150	0.468		−1.303	0.000	−0.408^*^
Rhythm		0.042	0.297		0.017	0.725		−0.094	0.700		−0.392	0.088	
Tempo		−0.074	0.070		0.020	0.673		−0.204	0.386		0.235	0.311	

*Beta-weights and p-values of the specific items included in the two linear and the two logistic regression analyses, performed separately for each general question (i.e., liking, adequateness, coherence, and profession). Dark cells represent significant effects (p < 0.05), gray cells represent marginal effects (0.05 < p < 0.10) and white cells correspond to non-significant predictors (p > 0.10). The columns “direction” include the coefficient correlations between the vocal expression and the general question. If the significance level of p < 0.05 is reached (marked with an asterisk), the sign of the coefficient correlation indicates the direction of the relation between vocal features and the general question. Note that non-significant correlations reflect the non-specificity of direction of the vocal features influencing the general rating. For Direction*:

* *p < 0.05*.

#### Adequateness

As with the liking rating, a significant regression equation was found for the adequateness scale, *F*_(20, 433)_ = 7.518, *p* < 0.001. Several items contributed in explaining 25.8% of the variance of adequateness ratings (see Table 4). Spearman correlations reveal that low adequateness was associated with continuous pitch changes, imprecise articulation and lengthened consonants. The items on average pitch and mode of phonation were not linearly correlated with the ratings on adequateness. Again, both directions were associated with low adequateness.

#### Coherence

The logistic regression model was statistically significant, $\chi_{\left(20\right)}^{2}$ = 142.65, *p* < 0.001. The model explained 37.3% (Nagelkerke *R*^2^) of the variance in coherence ratings and correctly classified 77.4% of cases. Increasing the ratings of pitch variability and range, timbre, faucal distance and vowel duration on the JAR-ness scale, increased the likelihood of perceiving the interpretation as incoherent (i.e., answer “no”). Incoherence was associated with lengthened vowels, a dark timbre and a wide pharynx as well as an inflected and monotone pitch variability and a wide and narrow pitch range.

#### Profession

The logistic regression model was statistically significant, $\chi_{\left(20\right)}^{2}$ = 96.48, *p* < 0.001. The model explained 26.2% (Nagelkerke *R*^2^) of the variance in profession ratings and correctly classified 74.0% of cases. “Too much” singing and a “too soft” sound of voice are associated with the fact that performers are perceived as singers whereas the choice of an actress (or speaker) was associated with “too much spoken” and a “too hard” vocal sound.

To conclude, the results show a significant agreement of the participants concerning specific items and a moderate agreement concerning general questions. Disregarding any specific interpretations, regression analyses highlight some particularly salient items when listening to and rating Schoenberg's Pierrot lunaire.

### Description of speechsong

**Figure 1:**
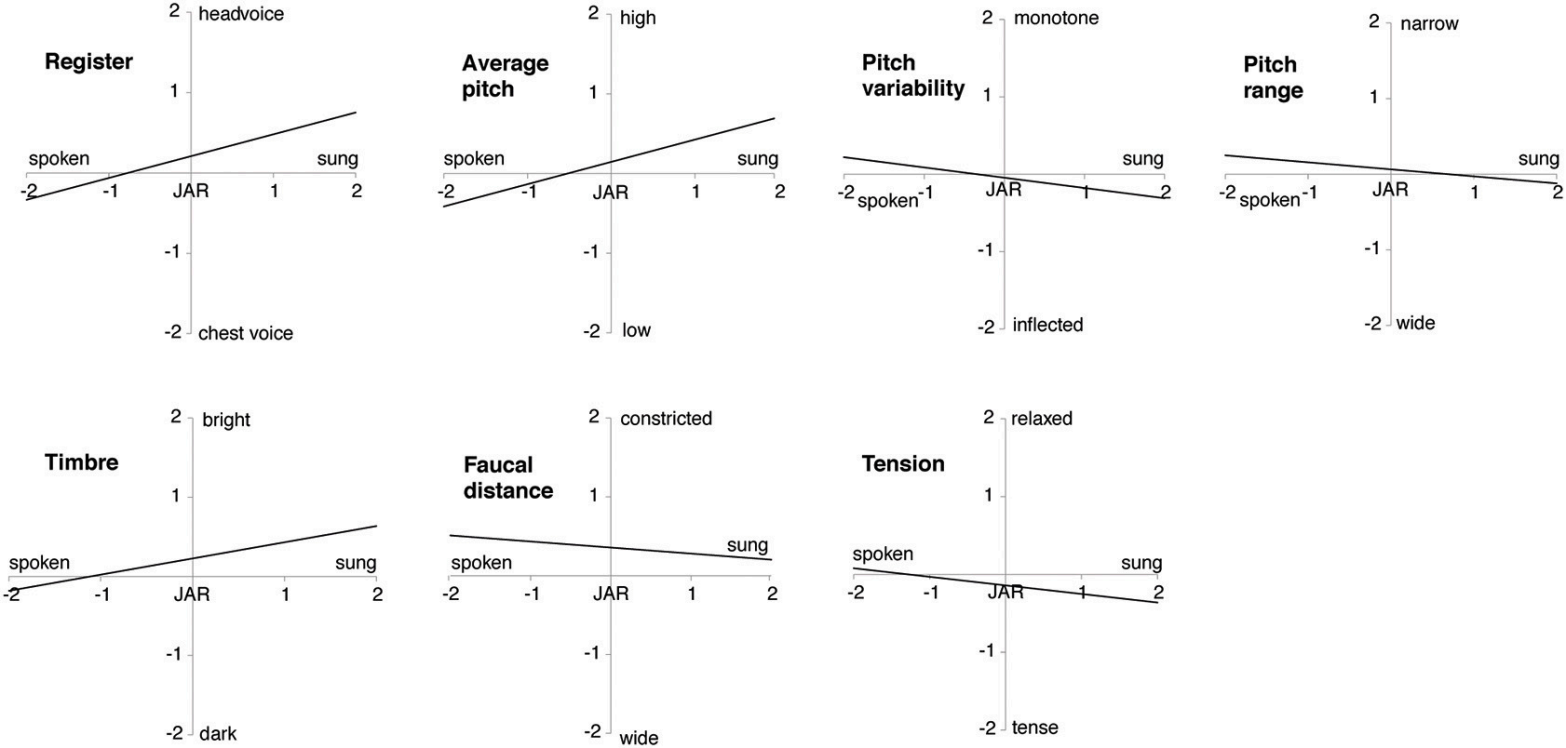
Features associated with song and speech. Illustration of the significant correlations between the different features (register, average pitch, pitch range, pitch variability, timbre, faucal distance, and tension; y-axis) and mode of phonation (spoken vs. sung, around “just about right,” JAR; x-axis).

The Spearman coefficient correlations highlight that singing correlates strongly with a high pitched voice (*r* = 0.513, *p* < 0.001), head voice (*r* = 0.313, *p* < 0.001) and a bright timbre (*r* = 0.335, *p* < 0.001). An inflected pitch contour (*r* = −0.208, *p* = 0.009), a wide pitch range (*r* = −0.188, *p* = 0.036), a wide faucal distance (*r* = −0.202, *p* = 0.019), and a tense phonation (*r* = −0.222, *p* = 0.008) might also be associated with singing. The feature of pitch changes showed only a tendency toward continuously changing pitch correlating with singing (*r* = 0.143, *p* = 0.067). For an illustration of the relationships, see Figure 1.

In light of the importance of this feature in the literature, it was of interest to investigate it further in order to get a more conclusive picture about the relation between the pitch changes and mode of phonation. Firstly, a Pearson Chi-Square test confirmed that the perception of pitch changes [$\chi_{\left(4\right)}^{2}$ = 22.240, *p* < 0.001] significantly differs depending on the perception of mode of phonation. Secondly, the cross table (Table 5) revealed that the expected count in three fields out of four is less than expected. In the row of “singing” ratings, sudden pitch changes show a count lower than expected, continuous pitch changes on the other hand show a count higher than expected. With respect to JAR-ratings, more continuous pitch changes are expected for JAR-phonation, pointing toward a reduced acceptance of pitch glides in speechsong performances.

**Table 5:** Cross-table pitch changes.

			**Pitch changes**			
			**Sudden**	**JAR**	**Continuous**	**Total**
**Mode of Phonation**	**Speaking**	Count Exp. count	27 19.8	23 37.1	32 25.1	82
	**JAR**	Count Exp. count	59 52.2	108 97.8	49 66.1	216
	**Singing**	Count Exp. count	35 48.0	94 90.1	71 60.9	199
Total		Count	120	225	152	497

*Cross-table (Count vs. Expected count) for the feature mode of phonation in comparison to pitch changes. The JAR-ratings were not included in the Chi-squared tests and are therefore depicted in gray*.

## Discussion

This study describes a new approach designed to clarify the vocal features used to categorize speech and song. By examining the perception of vocal expression when listening to several interpretations of speechsong, which is neither typical for speech nor song, we focused on the perceptual impressions of listeners.

### Characteristics of mode of phonation

The present approach and material allow for review and extension of existing proposals on the differences between song and speech, which until now have been mainly described with regard to pitch patterns of speech prosody and sung melody (e.g., discrete pitches and gliding pitch patterns, range and interval size). The current study allows for examination of several aspects of pitch and confirms typical behaviors in singing, such as a wider pitch range, high pitched voice and an inflected pitch contour (e.g., Patel, 2008; Zatorre and Baum, 2012; Mecke et al., 2016). Interestingly, the often mentioned difference in pitch changes was not supported. On the contrary, continuous pitch changes show a higher count than expected for singing and not speaking, suggesting that exaggerating the pitch glides leads to the impression of singing and does not enhance the impression of speaking. As a consequence, the pitch glides do not seem to be a stable indicator for speech. Depending on how pronounced the glides are produced, the perception might shift toward singing.

In addition to the usual pitch related features, the present study revealed that mode of phonation is associated with more features of vocal expression. As expected, head voice was associated with singing, but most frequently, if mode of phonation was just about right, the register was likewise just about right. Chest voice was underrepresented in the current material, and the combination of “too spoken” and “too much head voice” (despite Schoenberg's comments on speaking in head voice) was almost non-existent—which might also reflect listeners' basic assumption of the impossibility of this occurrence. According to our exploratory study, other features such as a tense phonation (reflecting high muscular activity), a wide faucal distance (reflecting a wide pharynx, lift of the soft palate and a low larynx), and a bright timbre (reflecting efficient use of resonance cavities), might also be associated with singing perception. Note that the association of spectral characteristics with singing is not surprising since they are particularly favored (if not specifically trained) in classical singing (Miller, 1986, 1996; Mitchell et al., 2003; Isherwood, 2013) and since listeners, even without formal training in music, are sensitive to such features (Larrouy-Maestri et al., 2017).

By asking for the assumed profession of the performer, our objective was to gain information on singing and speaking indirectly. As revealed by the logistic regression, singers and speakers were divided by soft and hard vocal onsets, respectively, but most importantly by mode of phonation (i.e., performers were perceived as singers if the performance was “too sung” and vice versa for speakers). This seems to reflect causality (i.e., attribution of a profession according to mode of phonation) but the hypothesis of an opposite relation (i.e., attribution of mode of phonation according to the profession) cannot be rejected. The fact that raters attribute the performance to a specific profession might lead to specific expectations (Falk et al., 2014; Vanden Bosch der Nederlanden et al., 2015) and thus bias the judgment toward speech or song. Future research controlling for the expected profession of the performer (e.g., by explicitly instructing the participants about the performer being an actor or a singer) would allow for clarification of the relationship observed between these two items.

Altogether, these findings extend the current knowledge on vocal features of speech and song, highlighting aspects of pitch, register, tension and timbre. Further research is encouraged to replicate the current findings by testing other “ecological” types of hybrid vocalizations, such as Rap, Jazz, or Musical Theater style singing or infant-directed speech. Phenomena such as speechsong are particularly interesting to the study of categorization processes, because they utilize our interest in ambiguous material in an artistic context, i.e., they esthetically challenge our internalized impressions of typical song and speech. By composers and performers playing with these expectations, researchers get information on the possible adjustments of certain vocal features. Finally, the identification of relevant features (i.e., associated with the perception of mode of phonation) paves the way to a better understanding of the perception and categorization processes of song and speech. The precise description of these features with acoustical analyses, and their systematic manipulations, will certainly clarify the categorization of vocal expression.

### Notes on “liking,” “adequateness,” and “coherence”

Despite the frequent use of such terminology, the concepts behind are either highly subjective or difficult to grasp, as reflected in the relatively low inter-rater agreement. In the current study, adequateness was meant to describe the degree in which the performer succeeded in meeting the composer's intentions. Despite the low agreement and the percentage of explained variance (25%), relevant items noted by the composer such as mode of phonation, average pitch and pitch changes predicted the ratings' variance. Unlike liking-ratings, which showed a higher percentage of explained variance (47%), the evaluation of adequateness might rely on additional features which were not proposed in the questionnaire. Alternatively, the low percentage of explained variance regarding adequateness ratings could be due to missing information about this peculiar piece prior to testing.

With the subjective question on liking, the current study tackles the question of vocal appreciation in the context of speechsong. While the dislike of features such as a high pitched voice, a thin and hard vocal sound and a constricted pharynx might be explained in a broader context of pathological vocal sounds, other features such as lengthened vowels, pitch changes and variability might be very specific to the context of the rated piece. This points to the relevance of investigating other (and maybe more liked) material to control for the appreciation of listeners when examining the perception of speechsong.

Finally, the concept of coherence reflects the listener's impression of the performance in general (which includes the flute and the voice). It can be interpreted as which features do not fit in with the listeners' expectations of the interpretation of the piece. An incoherent interpretation was associated with a monotone and inflected pitch contour, a wide and narrow pitch range and shortened vowels. Notably, these features are set by the musical score, which might mean that listeners base their expectations on what is typically confined by the sheet music.

### Notes on the questionnaire

The questionnaire to evaluate features of vocal-articulatory expression was adapted to describe vocal performances producing speechsong. The questionnaire is an attempt to cover vocal expression with several items to achieve detailed descriptions of listeners' impressions. This tool might not replace acoustical analyses of vocal features but provides the information required (i.e., perception of vocal expression) for further investigation of relevant acoustical features. The high reliability of the raters with regard to the features of vocal expression suggests that raters understood the items in this specific context. Also, the JAR-scale, implemented to give a midpoint that would reflect their acceptance of the features in the given situation, provides useful indications regarding listeners' judgments beyond a pure description of the material. Concluding from the high agreement among judges, despite the variability in terms of formal musical background, this finding supports that the adaptation of the questionnaire was successful and is adequate to describe vocal expression in speechsong. From these results one can assume that an internal validation of the questionnaire might be successful, which would require systematically manipulated stimuli including acoustical analyses as well as comparison with other questionnaires. Further evidence that the questionnaire fulfilled its purpose is the result that the ratings actually relate to the context information given by the experimenter.

The chosen questionnaire is meant to be a dynamic tool that can be adapted to different situations and listeners. Its use in the present form implies listeners' expertise in auditory description of voices (more due to the labeling of features than to perception itself) and thus limits its application to a specific group of participants. However, it could be adapted to lay listeners by providing additional instructions. Here, the features lay listeners are able to evaluate need to be investigated and are at the same time relevant for the discrimination of song and speech. The ratings by voice experts should be used as a baseline. Therefore, this tool seemed to be particularly relevant in the present context and might be used in future research on different vocal material.

## Conclusion

By examining listeners' perception of Schoenberg's Pierrot lunaire with regard to several features of vocal-articulatory expression, the present study highlights the features influencing the impression of song and speech in ecologically valid material. Keeping in mind the limitations due to the peculiar character of the piece under study, we observed the relevance of pitch, register, tension, and timbre. Besides clarifying the vocal features leading listeners' perception of a vocalization as being speech or song, our findings support the adequacy of both the chosen ambiguous material and the proposed questionnaire in investigating speech/song categorization. Also, this approach paves the way to further studies using other hybrid material as well as acoustically controlled manipulations of sounds to precisely define the acoustical characteristics driving speech and song perception and therefore to better understand the similarities/differences between music and language perception.

## Author contributions

JM conceptualized and conducted the research. JM and PL analyzed the data and wrote the article.

### Conflict of interest statement

The authors declare that the research was conducted in the absence of any commercial or financial relationships that could be construed as a potential conflict of interest.
